# Supplementary material for: A Likelihood Approach for Real-Time Calibration of Stochastic Compartmental Epidemic Models
Source: PLoS Comput Biol. 2017 Jan 17;13(1):e1005257. doi: 10.1371/journal.pcbi.1005257 (PMC5240920; doi:10.1371/journal.pcbi.1005257)
Supplement: S1 File — (TAR.GZ) [file pcbi.1005257.s014.tar.gz › HSPH_Online-SI-Revision/IPoi09/n1-mild/n1-mild_BENCH_table-8.pdf]

1	R0	{1.27677, 7.89375}	{1.79464, 13.65}	{{1.39507, 7.47062}, {2.22731, 18.788}}	{40.6926, 76.0965}	inf	9.	29.2778	{3., 69.}	252.469	0.497672	newly	11.	16.4815	{2., 40.}	102.694	0.402255	Reff	1.2423	1.72039	{1.3884, 2.05756}	39.351	ar	400.	705.185	{576., 845.}	83.2963
2	R0	{1.33231, 10.5259}	{1.84352, 10.2044}	{{1.68297, 8.49961}, {1.99822, 12.2033}}	{38.3703, 15.4437}	inf	43.	98.5962	{39., 162.}	135.107	0.342035	newly	32.	60.8654	{28., 94.}	99.2188	0.288659	Reff	1.07517	0.85639	{0.548278, 1.26678}	30.1964	ar	467.	754.904	{665., 821.}	61.6496
3	R0	{1.2273, 6.76944}	{2.20402, 3.27459}	{{1.4023, 1.89883}, {2.90743, 4.62276}}	{79.8418, 51.6269}	inf	19.	63.42	{0., 202.}	331.053	1.40281	newly	19.	157.79	{0., 402.}	820.474	1.62754	Reff	1.19048	1.85442	{1.24384, 2.52695}	58.0602	ar	314.	448.42	{1., 921.}	133.917
4	R0	{1.41215, 17.5742}	{1.60817, 11.2458}	{{1.2743, 5.50601}, {2.00043, 17.5151}}	{19.5764, 37.3883}	inf	19.	46.7368	{10., 98.}	167.867	0.504531	newly	6.	31.0526	{3., 57.}	434.211	0.739442	Reff	1.34578	1.39514	{0.9606, 1.90773}	20.7732	ar	465.	624.447	{388., 798.}	46.3667
5	R0	{1.42222, 6.15886}	{1.48395, 4.76047}	{{1.24751, 2.5364}, {1.95355, 8.88568}}	{14.8529, 48.1344}	inf	13.	45.2258	{7., 86.}	274.69	0.656524	newly	24.	62.6774	{8., 118.}	190.995	0.58695	Reff	1.34115	1.36544	{0.87266, 2.13274}	29.3438	ar	490.	571.903	{138., 882.}	43.2456
6	R0	{1.24363, 9.95596}	{1.95837, 11.5287}	{{1.15409, 5.21967}, {2.83341, 18.4504}}	{60.1731, 42.7676}	inf	3.	2.05	{0., 5.}	81.	0.692235	newly	3.	2.61	{0., 4.}	115.	0.905717	Reff	1.23741	1.95139	{1.15063, 2.83058}	60.3437	ar	411.	335.55	{1., 907.}	98.5377
7	R0	{1.36478, 5.96056}	{1.94753, 5.4867}	{{1.27121, 2.01465}, {2.82914, 9.34414}}	{46.392, 38.6336}	inf	14.	36.0667	{0., 87.}	209.762	0.758598	newly	23.	61.6667	{0., 145.}	212.899	0.796365	Reff	1.32384	1.8813	{1.22553, 2.61511}	45.8302	ar	484.	604.25	{4., 918.}	67.8065
8	R0	{1.36335, 17.6429}	{1.20987, 14.6874}	{{1.0306, 9.66506}, {1.36512, 18.9003}}	{12.6826, 20.4002}	inf	13.	8.17391	{1., 20.}	75.2508	0.663586	newly	3.	3.73913	{0., 10.}	108.696	0.596143	Reff	1.33472	1.19451	{1.02681, 1.38091}	12.6619	ar	484.	253.652	{13., 557.}	55.1383
9	R0	{1.26575, 5.28707}	{1.47804, 7.49335}	{{1.40191, 5.7404}, {1.62191, 10.1332}}	{16.7715, 41.9678}	inf	7.	44.7179	{19., 69.}	542.491	0.758945	newly	8.	40.7949	{21., 62.}	413.782	0.678561	Reff	1.13918	1.069	{0.822921, 1.40999}	16.1235	ar	472.	547.513	{399., 680.}	21.507
10	R0	{1.42942, 9.49905}	{1.72377, 10.7942}	{{1.40191, 5.7404}, {2.01705, 15.0173}}	{21.0592, 30.2296}	inf	25.	86.8519	{21., 156.}	257.926	0.505275	newly	22.	52.037	{21., 81.}	147.138	0.384538	Reff	1.26932	1.12494	{0.732539, 1.68034}	27.2168	ar	468.	697.741	{538., 827.}	50.3166
11	R0	{1.46827, 15.0983}	{1.22942, 9.76803}	{{1.10202, 5.72868}, {1.49434, 16.5722}}	{17.3424, 41.5078}	inf	7.	12.7727	{1., 28.}	130.519	0.416986	newly	4.	8.27273	{1., 20.}	150.	0.455456	Reff	1.43009	1.18997	{0.956379, 1.50967}	19.454	ar	495.	363.727	{54., 652.}	41.8916
12	R0	{1.25751, 13.279}	{1.21257, 6.46482}	{{1.11523, 3.74835}, {1.32613, 9.33397}}	{7.3228, 52.7352}	inf	9.	18.7083	{4., 35.}	131.944	0.416292	newly	6.	19.6667	{4., 33.}	244.444	0.550063	Reff	1.20847	1.02567	{0.829731, 1.24043}	18.5092	ar	366.	353.917	{191., 490.}	27.8233
13	R0	{1.3425, 9.16361}	{1.49652, 5.40934}	{{1.454, 4.47101}, {1.54465, 5.4396}}	{11.4725, 41.2065}	inf	36.	37.2857	{11., 69.}	47.8836	0.319565	newly	33.	42.0476	{10., 69.}	60.8947	0.332038	Reff	1.12233	0.868743	{0.657047, 1.23726}	26.9471	ar	442.	549.071	{452., 658.}	31.4641
14	R0	{1.18936, 6.20583}	{2.24524, 13.1084}	{{1.67405, 8.12461}, {2.86501, 18.1089}}	{88.7773, 115.205}	inf	20.	35.7119	{3., 74.}	121.271	0.424962	newly	20.	24.9831	{3., 58.}	79.322	0.390347	Reff	1.1287	2.1331	{1.66999, 2.67807}	88.9869	ar	382.	814.373	{720., 929.}	116.967
15	R0	{1.27137, 9.78868}	{1.52373, 5.37073}	{{1.24751, 2.5364}, {2.33995, 13.5694}}	{20.7814, 57.4642}	inf	14.	38.5161	{4., 67.}	209.217	0.58528	newly	12.	48.8065	{2., 116.}	336.828	0.716134	Reff	1.20017	1.49245	{0.869514, 2.36526}	39.7867	ar	496.	579.806	{156., 908.}	47.1124
16	R0	{1.23102, 13.6452}	{2.00963, 14.961}	{{1.52414, 9.30931}, {2.27287, 19.1478}}	{63.2494, 23.989}	inf	23.	55.0189	{8., 100.}	168.089	0.464643	newly	11.	28.7358	{2., 61.}	191.767	0.53725	Reff	1.16085	1.82702	{1.46662, 2.17967}	57.935	ar	445.	784.302	{692., 876.}	79.7583
17	R0	{1.27213, 13.7452}	{1.98882, 14.3406}	{{1.44641, 8.4085}, {2.51417, 19.5287}}	{56.7948, 26.0248}	inf	8.	43.5902	{0., 119.}	492.828	0.89495	newly	5.	23.7377	{0., 62.}	424.59	0.848142	Reff	1.2505	1.85916	{1.44058, 2.23516}	48.6731	ar	367.	650.23	{4., 884.}	112.766
18	R0	{1.25318, 11.2151}	{1.31175, 7.46884}	{{1.24271, 5.50601}, {1.39882, 9.33397}}	{5.52298, 34.9585}	inf	12.	23.5714	{3., 55.}	130.952	0.386777	newly	5.	19.7857	{2., 51.}	312.857	0.554313	Reff	1.17924	1.08401	{0.866072, 1.33717}	13.7028	ar	416.	402.571	{138., 568.}	28.1765
19	R0	{1.46006, 8.11496}	{1.80679, 13.6257}	{{1.24271, 7.81552}, {2.29738, 18.9086}}	{28.963, 70.9631}	inf	12.	8.91228	{0., 25.}	74.5614	0.674748	newly	15.	6.15789	{0., 19.}	78.1287	0.935848	Reff	1.43232	1.82679	{1.36144, 2.31385}	29.9686	ar	496.	529.281	{3., 872.}	62.2347
20	R0	{1.14273, 6.71261}	{1.27238, 6.25528}	{{1.07662, 3.02112}, {1.66328, 11.8799}}	{15.0073, 45.2997}	inf	10.	17.8	{3., 38.}	118.	0.376253	newly	11.	17.56	{2., 35.}	109.818	0.414683	Reff	1.10273	1.30769	{0.966805, 1.73061}	24.1417	ar	362.	439.52	{147., 771.}	61.3481
21	R0	{1.42432, 8.23905}	{1.40639, 2.56127}	{{1.4031, 2.55684}, {1.4031, 2.55684}}	{1.45262, 68.9131}	inf	12.	9.34483	{0., 29.}	83.6207	0.91897	newly	16.	18.7586	{0., 66.}	118.534	1.00099	Reff	1.21067	0.726258	{0.534581, 0.895178}	40.012	ar	405.	509.724	{383., 619.}	29.57
22	R0	{1.29386, 17.863}	{1.68477, 13.3953}	{{1.21937, 6.16288}, {2.15611, 18.9003}}	{33.2878, 27.2726}	inf	15.	20.5111	{3., 51.}	92.4444	0.466647	newly	3.	12.6	{0., 35.}	348.148	0.655403	Reff	1.26151	1.62289	{1.17426, 2.04843}	31.9723	ar	488.	578.267	{34., 806.}	52.5592
23	R0	{1.39274, 13.0167}	{1.60901, 8.405}	{{1.44485, 5.65591}, {1.81743, 11.799}}	{15.6234, 36.2319}	inf	20.	50.2708	{4., 119.}	189.688	0.555256	newly	9.	30.2917	{3., 62.}	265.741	0.607997	Reff	1.22283	0.724337	{0.512311, 0.975884}	41.5743	ar	475.	657.833	{515., 761.}	39.1228
24	R0	{1.32178, 15.8991}	{1.56203, 11.8407}	{{1.24271, 6.05643}, {1.95859, 18.4377}}	{20.8903, 31.0042}	inf	13.	32.475	{0., 70.}	190.577	0.616591	newly	5.	20.2	{0., 45.}	335.	0.68582	Reff	1.27023	1.46711	{1.05963, 1.76547}	22.7898	ar	471.	531.425	{23., 817.}	51.173
25	R0	{1.37568, 17.8427}	{1.66972, 12.5918}	{{1.42446, 7.81552}, {1.88811, 16.1198}}	{21.4483, 30.1792}	inf	15.	64.122	{23., 106.}	333.659	0.595516	newly	9.	36.5122	{14., 57.}	310.027	0.570148	Reff	1.2615	1.32102	{1.03036, 1.6688}	16.2734	ar	398.	655.634	{561., 768.}	68.9974
26	R0	{1.20993, 6.4656}	{1.89038, 13.2816}	{{1.41695, 7.40677}, {2.25962, 18.743}}	{56.2386, 107.201}	inf	16.	47.7308	{7., 99.}	221.635	0.511988	newly	14.	28.1731	{5., 61.}	133.379	0.424206	Reff	1.13007	1.74001	{1.35451, 2.07431}	54.9573	ar	345.	737.827	{596., 865.}	117.598
27	R0	{1.20548, 13.8003}	{1.42189, 14.0643}	{{1.19093, 8.13911}, {1.60085, 19.1171}}	{18.6589, 24.5135}	inf	23.	25.9714	{7., 53.}	57.6398	0.291237	newly	9.	13.1143	{2., 30.}	86.9841	0.377967	Reff	1.15003	1.38454	{1.09868, 1.56687}	23.3737	ar	350.	546.657	{394., 680.}	63.3551
28	R0	{1.3176, 14.6594}	{1.32536, 12.4325}	{{1.06501, 6.18069}, {1.75814, 18.8819}}	{15.2165, 28.0171}	inf	4.	8.	{0., 17.}	159.524	0.646779	newly	1.	4.7381	{0., 12.}	426.19	0.734625	Reff	1.30442	1.32856	{1.04348, 1.6665}	15.5389	ar	362.	298.262	{3., 673.}	71.6193
29	R0	{1.28202, 6.23047}	{1.38036, 7.00517}	{{1.22516, 3.79186}, {1.68954, 12.6816}}	{10.4827, 37.5475}	inf	7.	34.6452	{10., 69.}	401.382	0.648102	newly	13.	32.2903	{11., 62.}	163.772	0.430629	Reff	1.22561	1.18534	{0.871621, 1.65619}	22.4421	ar	391.	513.129	{302., 752.}	47.1248
30	R0	{1.28177, 8.83252}	{1.9327, 11.4724}	{{1.20105, 3.78882}, {2.59755, 18.4249}}	{54.2397, 58.2815}	inf	15.	26.4528	{3., 59.}	127.421	0.491091	newly	15.	17.8302	{2., 34.}	80.7547	0.460922	Reff	1.24332	2.06381	{1.53187, 2.64095}	66.2795	ar	427.	741.	{428., 916.}	90.8753
31	R0	{1.35886, 5.97708}	{2.256, 14.6169}	{{1.77826, 9.44073}, {2.7811, 19.6712}}	{66.0213, 145.308}	inf	20.	46.9667	{7., 90.}	161.667	0.426543	newly	20.	28.95	{4., 57.}	78.75	0.334218	Reff	1.27733	2.12961	{1.63422, 2.52622}	67.7405	ar	491.	847.933	{728., 919.}	72.6952
32	R0	{1.27601, 17.9804}	{1.56748, 15.4065}	{{1.31707, 10.7271}, {1.79789, 19.4054}}	{23.3246, 18.2695}	inf	14.	35.5306	{0., 82.}	199.563	0.653748	newly	3.	16.7755	{0., 38.}	494.558	0.801557	Reff	1.24028	1.45106	{1.18915, 1.72954}	19.9958	ar	445.	534.612	{21., 765.}	55.4231
33	R0	{1.3368, 11.3235}	{1.68689, 11.5497}	{{1.2743, 5.40945}, {2.19504, 17.2142}}	{28.2359, 35.356}	inf	17.	32.3556	{5., 64.}	124.575	0.437321	newly	9.	23.3778	{4., 48.}	185.926	0.454517	Reff	1.27531	1.61949	{1.14954, 2.00433}	30.7611	ar	417.	663.311	{417., 835.}	68.1481
34	R0	{1.21021, 11.3635}	{1.28405, 3.86002}	{{1.22544, 3.07157}, {1.31748, 4.25564}}	{6.10126, 66.0314}	inf	11.	15.52	{6., 30.}	78.1818	0.282383	newly	11.	23.96	{6., 43.}	141.091	0.375253	Reff	1.12671	0.847607	{0.683569, 1.09351}	25.1931	ar	327.	414.4	{327., 558.}	35.3394
35	R0	{1.53382, 10.8866}	{1.73929, 14.2878}	{{1.44641, 10.2395}, {2.11476, 19.1478}}	{16.6553, 36.24}	inf	11.	16.3673	{1., 38.}	111.503	0.494785	newly	11.	9.10204	{0., 21.}	74.7681	0.574001	Reff	1.49547	1.73743	{1.48408, 2.04074}	18.0387	ar	476.	622.367	{17., 834.}	58.0604
36	R0	{1.20423, 6.83844}	{2.22566, 14.9034}	{{1.61188, 9.44073}, {2.78218, 19.6625}}	{84.9678, 119.419}	inf	6.	14.8462	{1., 34.}	183.333	0.491457	newly	7.	9.49231	{1., 23.}	100.659	0.478121	Reff	1.18255	2.15962	{1.59093, 2.65306}	83.2715	ar	416.	743.215	{248., 920.}	98.199
37	R0	{1.43629, 10.1548}	{1.28045, 3.86008}	{{1.22544, 3.07157}, {1.31748, 4.25564}}	{11.9934, 63.4688}	inf	10.	16.7308	{0., 23.}	147.308	0.634583	newly	5.	16.1538	{0., 37.}	269.231	0.697644	Reff	1.34006	0.842111	{0.681056, 1.15292}	37.18	ar	367.	401.462	{208., 496.}	26.5982
38	R0	{1.37504, 8.93428}	{2.0911, 11.9232}	{{1.25656, 3.82585}, {2.65407, 16.9461}}	{54.9385, 52.7597}	inf	15.	31.8393	{1., 79.}	166.548	0.608281	newly	14.	23.0714	{0., 64.}	123.98	0.572284	Reff	1.32829	2.05404	{1.5889, 2.53035}	56.2168	ar	483.	714.304	{21., 907.}	73.2365
39	R0	{1.51637, 9.36742}	{1.30285, 3.02945}	{{1.24751, 2.5364}, {1.36035, 3.26445}}	{14.0807, 67.6597}	inf	28.	18.88	{0., 35.}	46.	0.530788	newly	20.	33.72	{0., 71.}	108.6	0.572316	Reff	1.37686	0.884823	{0.691801, 1.09019}	35.7363	ar	441.	407.72	{228., 511.}	21.6599
40	R0	{1.23658, 9.82869}	{2.27406, 9.48744}	{{1.49267, 4.24222}, {2.94803, 13.5542}}	{83.8988, 27.2377}	inf	23.	65.6207	{11., 141.}	211.244	0.486891	newly	14.	62.0345	{9., 144.}	355.665	0.594024	Reff	1.18712	2.11727	{1.44228, 2.69383}	78.4192	ar	414.	836.828	{659., 942.}	106.372
41	R0	{1.24247, 5.4242}	{2.09452, 10.8078}	{{1.22544, 3.07157}, {2.87181, 17.2484}}	{70.7603, 115.539}	inf	9.	5.95455	{1., 15.}	61.4478	0.539312	newly	11.	6.42424	{0., 15.}	74.9311	0.721872	Reff	1.22135	2.19088	{1.41765, 2.84022}	80.3457	ar	345.	664.091	{3., 930.}	130.927
42	R0	{1.28208, 4.40223}	{2.15376, 10.1295}	{{1.31023, 3.79186}, {2.88523, 15.3124}}	{69.239, 137.556}	inf	3.	16.3148	{2., 31.}	462.346	0.69916	newly	6.	15.7037	{1., 39.}	195.679	0.51988	Reff	1.26028	2.29284	{1.54684, 2.8225}	81.9304	ar	339.	766.963	{441., 931.}	144.532
43	R0	{1.56773, 6.50474}	{2.00741, 9.08109}	{{1.24751, 3.3318}, {2.7943, 14.5901}}	{38.5021, 63.3637}	inf	6.	3.39	{0., 10.}	81.8333	0.865596	newly	5.	7.92	{0., 25.}	178.	1.01827	Reff	1.55205	1.99566	{1.24626, 2.74498}	38.8487	ar	498.	374.94	{1., 909.}	81.241
44	R0	{1.10489, 6.01941}	{1.32815, 6.41505}	{{1.22516, 3.79186}, {1.52414, 9.52864}}	{20.2067, 26.989}	inf	18.	30.9545	{2., 75.}	118.939	0.500175	newly	22.	30.1818	{3., 59.}	93.3884	0.489273	Reff	1.03418	1.0388	{0.679414, 1.2992}	19.5821	ar	346.	434.045	{51., 631.}	53.1398
45	R0	{1.25888, 14.1926}	{1.42988, 6.94052}	{{1.3618, 4.98002}, {1.54207, 8.77207}}	{13.5834, 51.0976}	inf	42.	37.561	{5., 69.}	49.9419	0.359234	newly	17.	33.9024	{5., 61.}	126.112	0.422403	Reff	1.09523	0.953135	{0.668644, 1.29084}	21.3572	ar	471.	519.976	{399., 624.}	20.9311
46	R0	{1.45292, 16.616}	{1.46157, 12.9354}	{{1.03515, 6.31117}, {1.83921, 18.5502}}	{17.1999, 26.5373}	inf	9.	9.23529	{0., 24.}	92.3747	0.810698	newly	4.	5.76471	{0., 13.}	132.353	0.797625	Reff	1.43258	1.48078	{1.12737, 1.87107}	17.5704	ar	494.	339.765	{2., 744.}	63.4675
47	R0	{1.22528, 16.6033}	{1.4207, 12.9145}	{{1.13176, 6.83219}, {1.75588, 19.1171}}	{20.1082, 27.0654}	inf	12.	10.6216	{1., 30.}	73.6486	0.536693	newly	3.	6.7027	{0., 18.}	166.667	0.490269	Reff	1.19955	1.429	{1.14063, 1.79525}	22.9096	ar	368.	398.811	{8., 750.}	73.2961
48	R0	{1.1669, 6.92715}	{1.55437, 9.51037}	{{1.38815, 6.71734}, {1.79436, 13.8428}}	{33.205, 38.9673}	inf	9.	62.973	{21., 128.}	599.7	0.754353	newly	10.	42.3243	{18., 64.}	323.243	0.574225	Reff	1.05138	1.16129	{0.831885, 1.5033}	22.01	ar	416.	629.946	{500., 756.}	52.7417
49	R0	{1.32749, 16.0887}	{1.52351, 12.493}	{{1.17434, 6.74711}, {1.85253, 19.0269}}	{19.336, 28.7733}	inf	11.	28.5526	{1., 58.}	200.239	0.602271	newly	7.	16.0263	{1., 33.}	175.564	0.560272	Reff	1.29165	1.48193	{1.12908, 1.81376}	21.3733	ar	353.	541.526	{28., 767.}	86.447
50	R0	{1.29636, 15.2609}	{1.98087, 16.3709}	{{1.68954, 12.272}, {2.2194, 19.5128}}	{52.8027, 15.6462}	inf	39.	70.0169	{18., 133.}	104.129	0.333138	newly	16.	34.4068	{10., 60.}	131.356	0.360196	Reff	1.16283	1.65017	{1.36964, 1.92567}	42.8718	ar	470.	769.288	{676., 859.}	66.8013
